# Supplementary material for: Alleviation of Collagen-Induced Arthritis by Crotonoside through Modulation of Dendritic Cell Differentiation and Activation
Source: Plants (Basel). 2020 Nov 10;9(11):1535. doi: 10.3390/plants9111535 (PMC7698099; doi:10.3390/plants9111535)
Supplement: Supplementary file 1 [file plants-09-01535-s001.zip › plants-994285-supplementary-proof/supplementary Figures.docx]

**Supplementary Materials:**


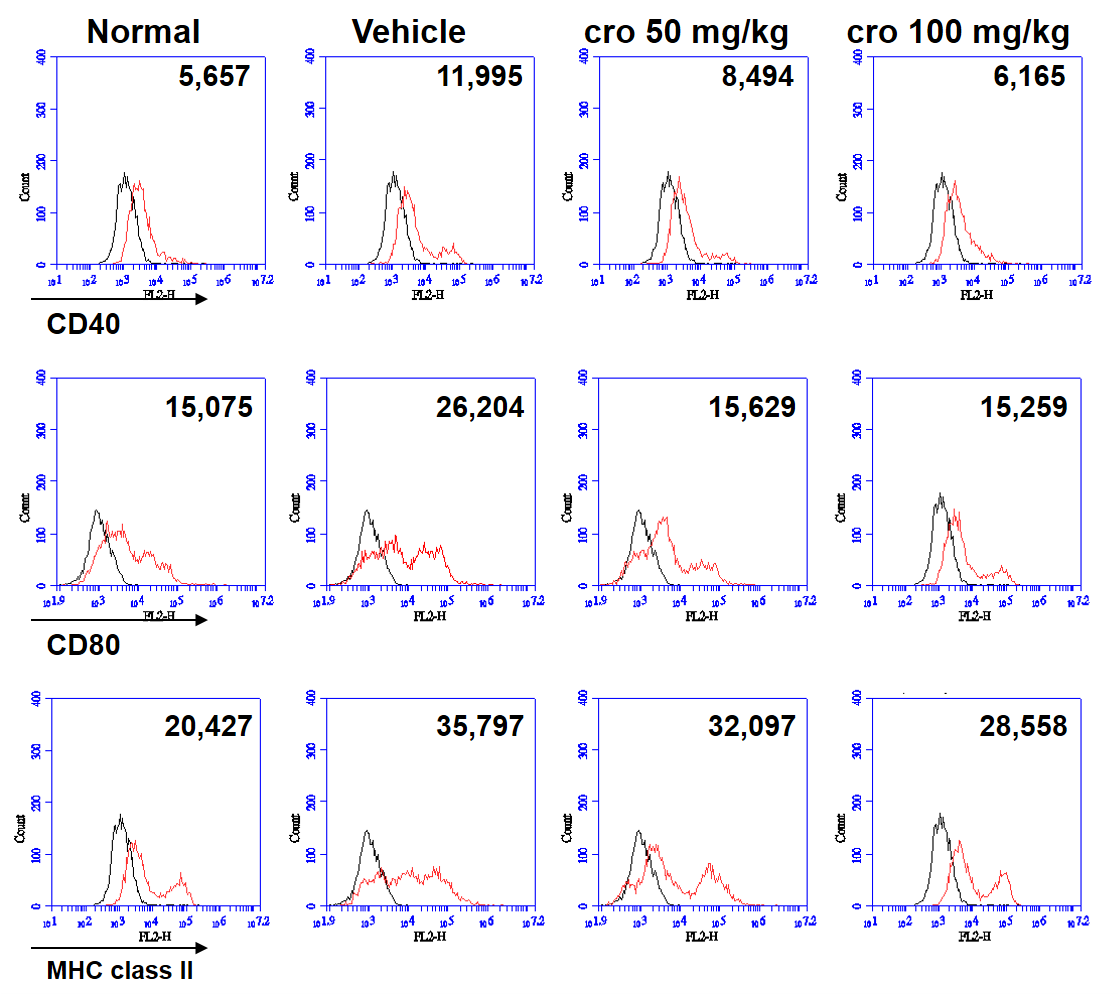


**Figure S1**. Representative bivariate graphs of co-stimulatory molecule, CD40, CD80, and MHC-II expressions of splenic CD11c^+^ DCs from CIA mice obtained on day 42 after CIA experiments.


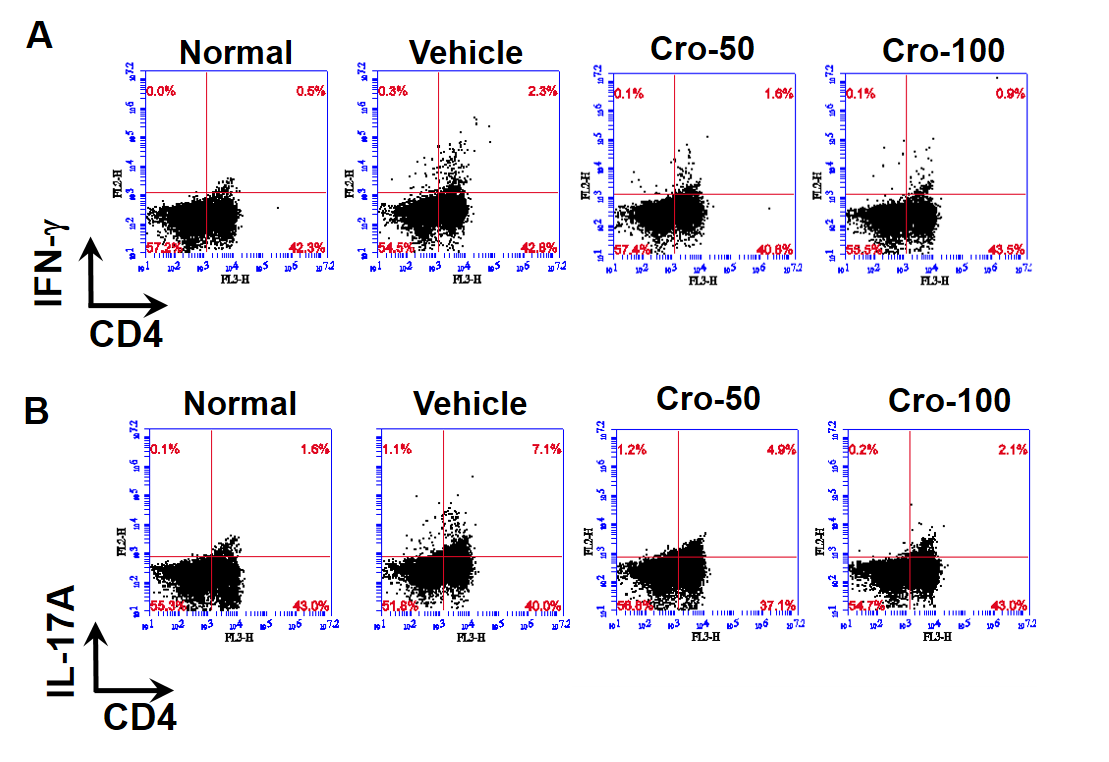


**Figure S2**. Representative bivariate graphs of (A) IFN-γ and (B) IL-17A expressed splenic CD4 T cells from one of six CIA mice after CII-stimulation and crotonoside treatments followed by analyzing with flow cytometry.


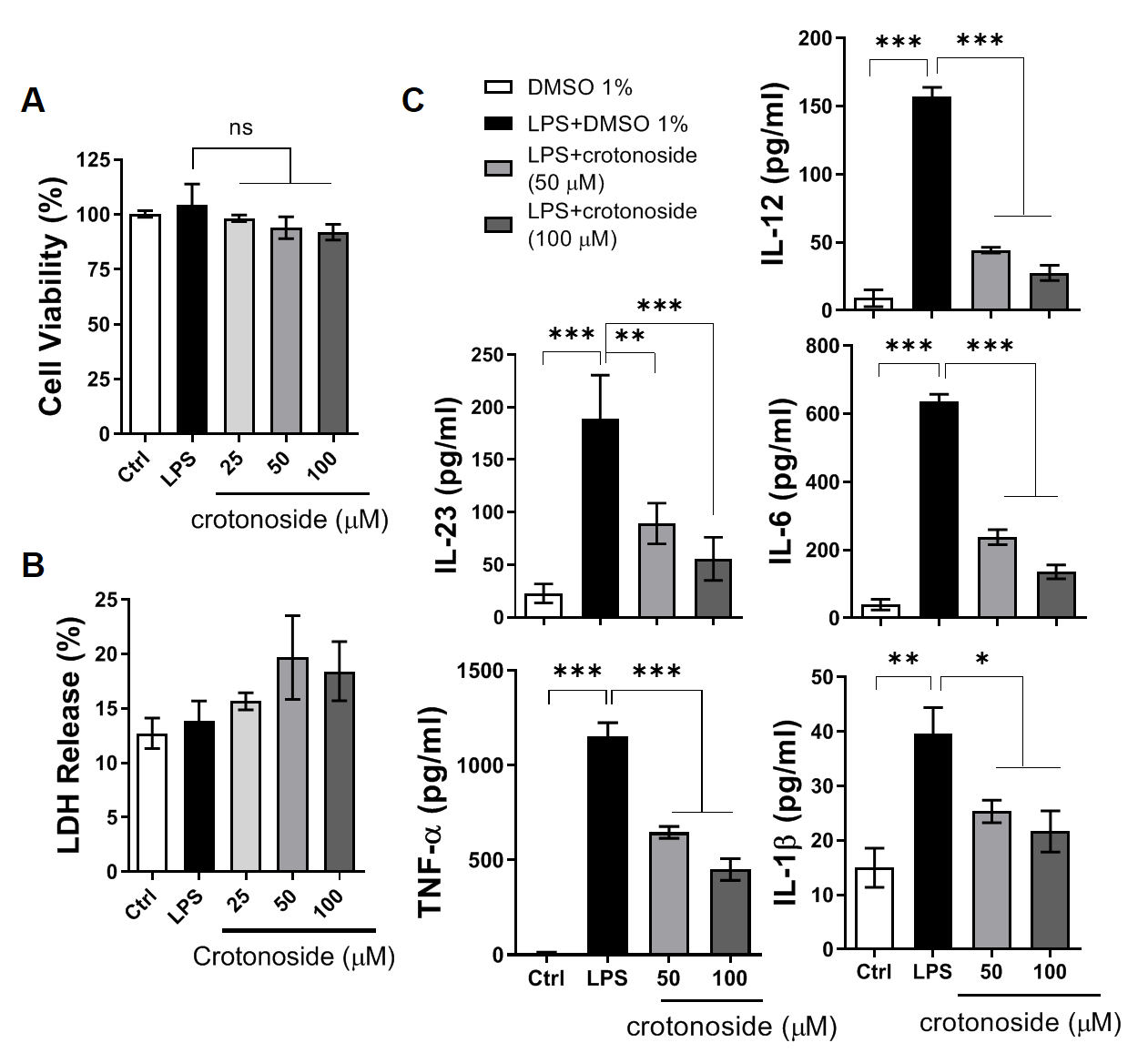


**Figure S3**. Cell viability and cytokine production of DCs after crotonoside treatment. LPS-stimulated, crotonoside-treated BMDCs, except DMSO mock control, were examined for (A) cell viability by CCK-8 (B) LDH assay and (C) cytokine productions by ELISA. Triplicate values represented as the mean ± SD from one of the three independent experiments with similar results. *P < 0.05, **P< 0.01, ***P < 0.001 versus LPS+DMSO-treated control, as determined by a one-way ANOVA with Dunnett test.


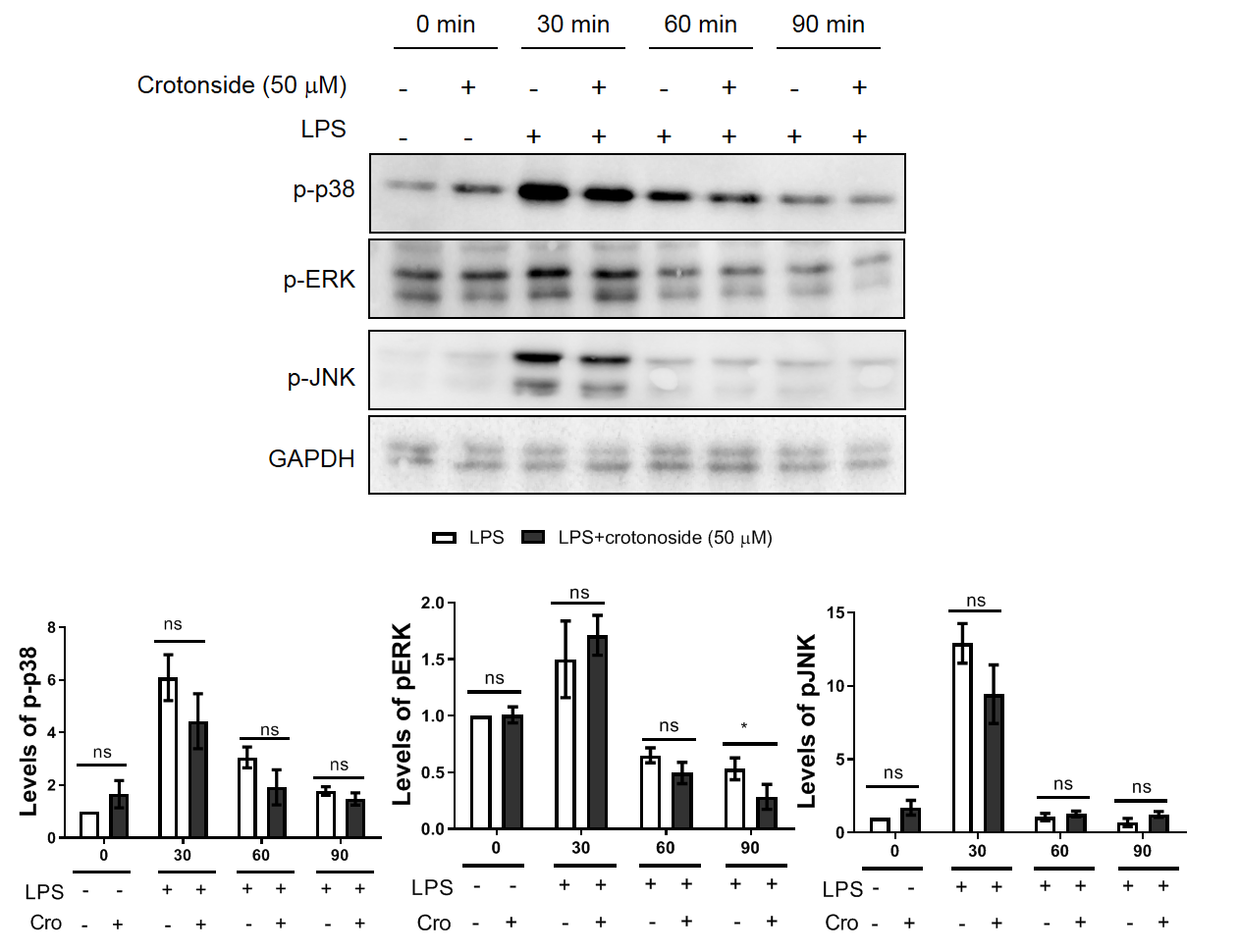


**Figure S4.** Phosphorylation of MAPKs in activated BMDCs. (A) Levels of phosphorylated p38, JNK, and ERK from LPS-stimulated BMDC cellular lysates at indicated time points with or without crotonoside (50 μM) were analyzed by Western blotting and quantified the intensity of bands in panel (B). Representative images from three independent experiments with similar results and GAPDH were used as loading controls. Values represented as the mean ± SD (n=3). *P < 0.05, **P< 0.01 versus LPS+DMSO-treated control at each time point, as determined by Students’ t test.
