# Supplementary figures and images for: Alleviation of Collagen-Induced Arthritis by Crotonoside through Modulation of Dendritic Cell Differentiation and Activation
Source: Plants (Basel). 2020 Nov 10;9(11):1535. doi: 10.3390/plants9111535 (PMC7698099; doi:10.3390/plants9111535)

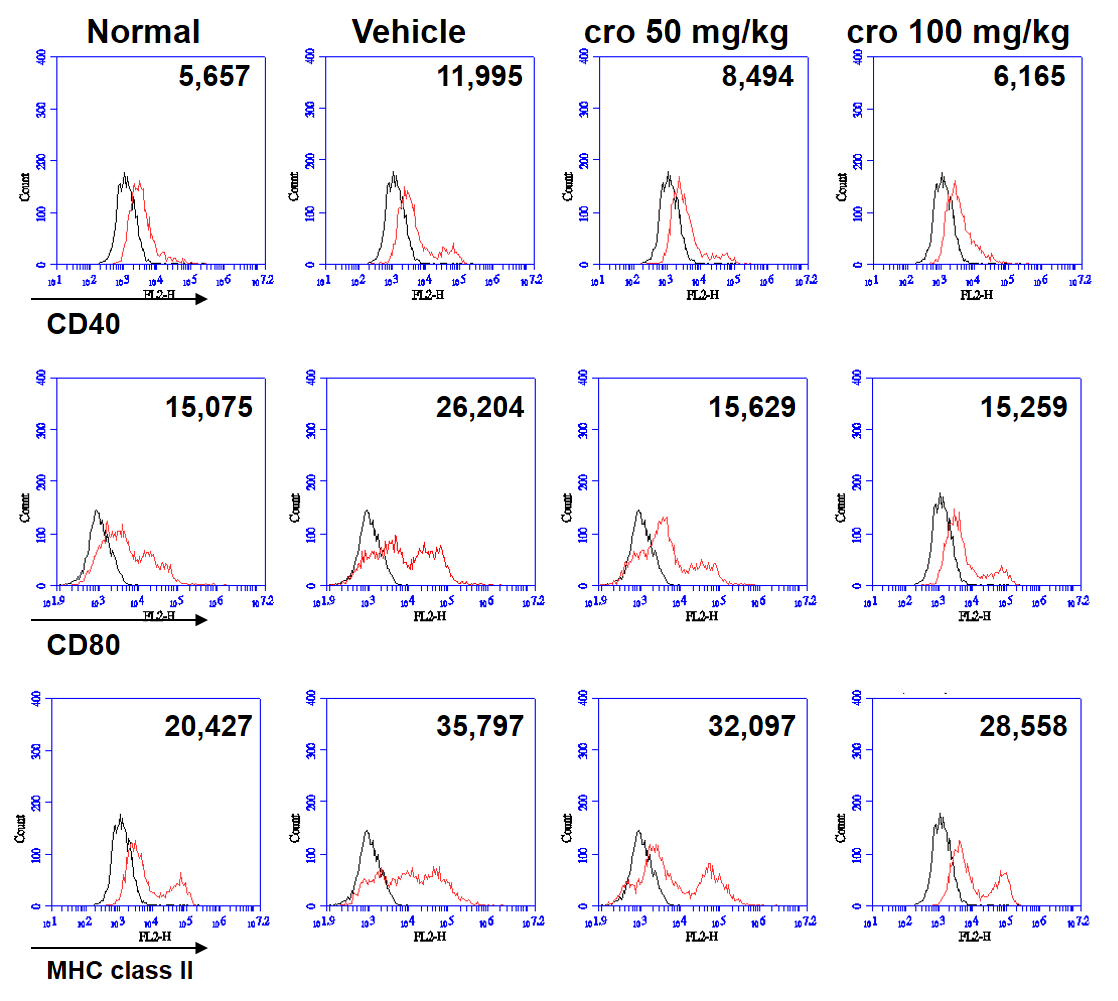

Supplement: Supplementary file 1 [file plants-09-01535-s001.zip › plants-994285-supplementary-proof/Suppl_Fig1.png]

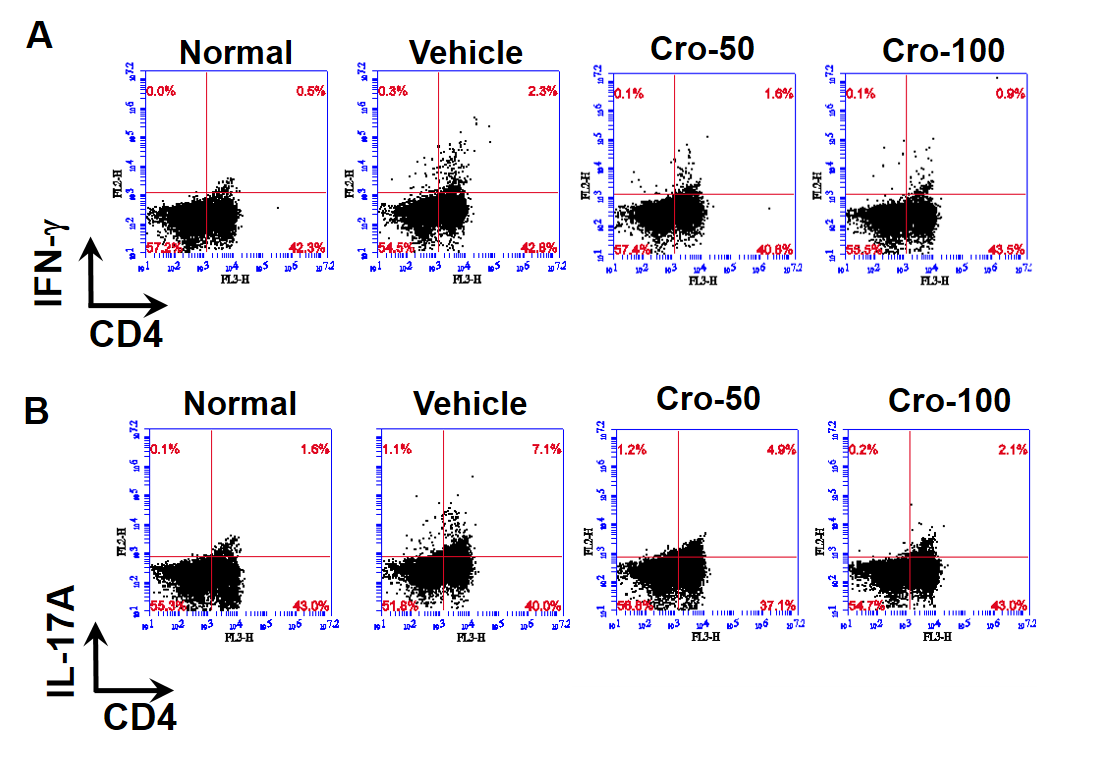

Supplement: Supplementary file 1 [file plants-09-01535-s001.zip › plants-994285-supplementary-proof/Suppl_Fig2.png]

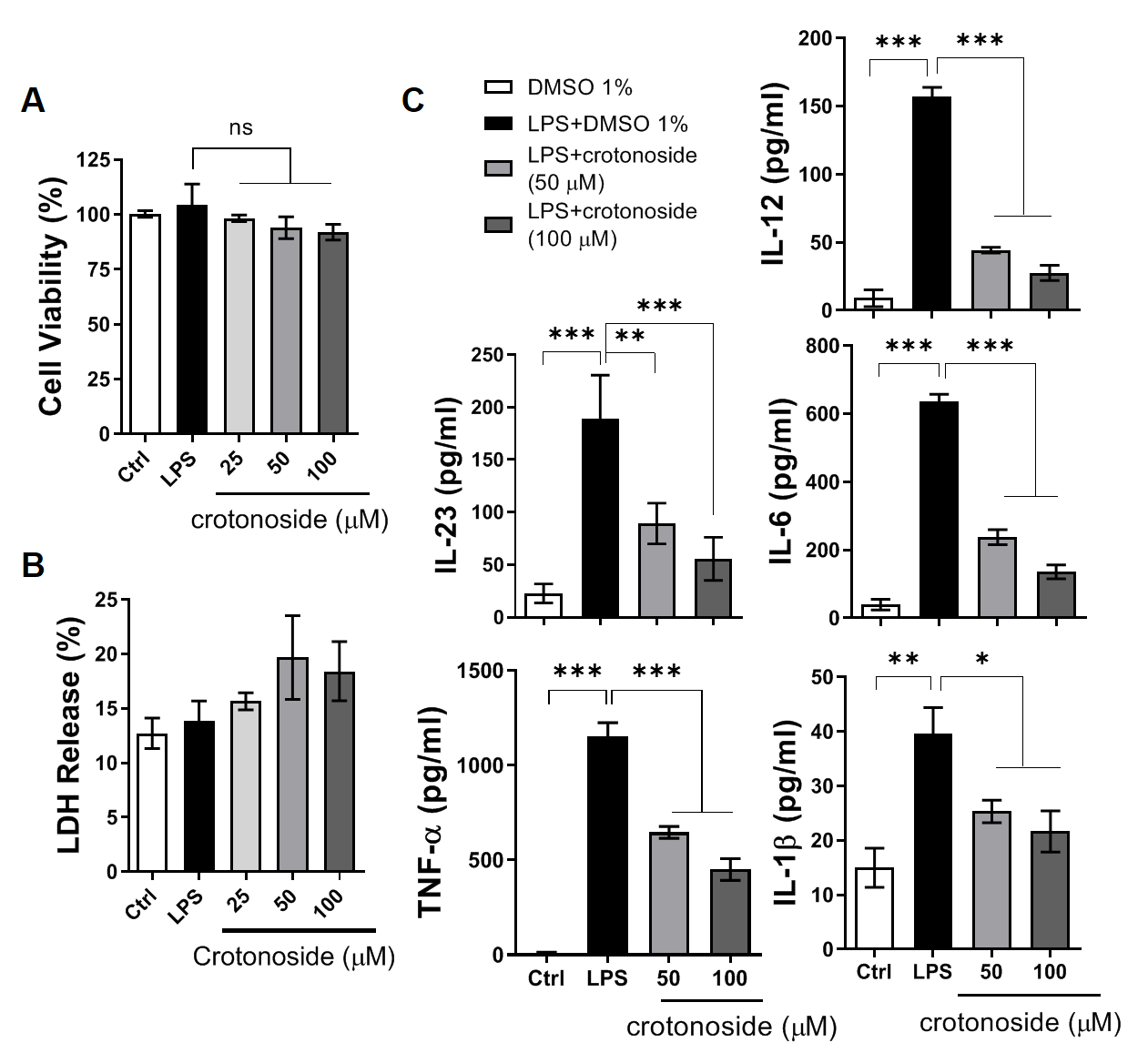

Supplement: Supplementary file 1 [file plants-09-01535-s001.zip › plants-994285-supplementary-proof/Suppl_Fig3.png]

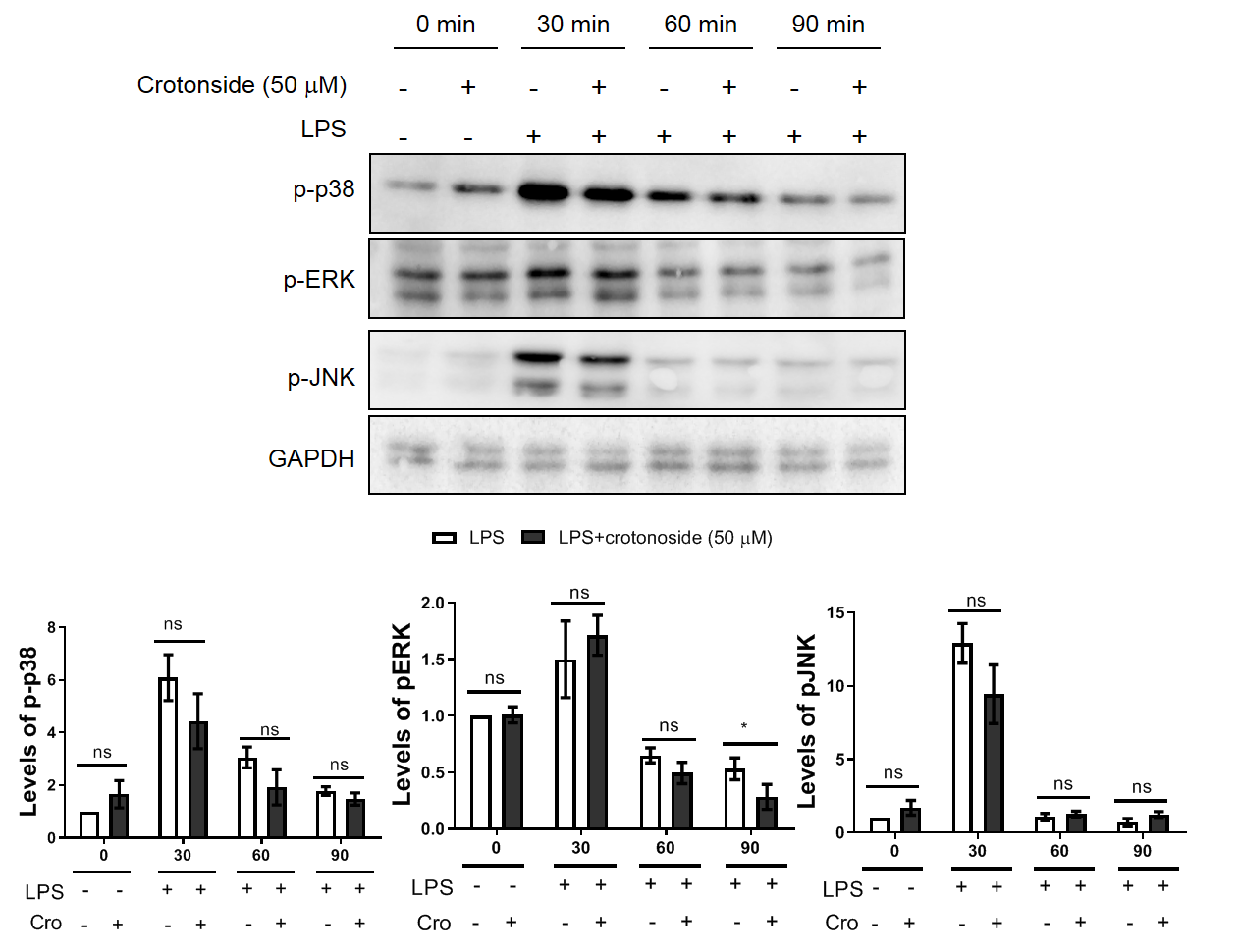

Supplement: Supplementary file 1 [file plants-09-01535-s001.zip › plants-994285-supplementary-proof/Suppl_Fig4.png]
